# Peripheral Glutamate Receptors Are Required for Hyperalgesia Induced by Capsaicin

**DOI:** 10.1155/2012/915706

**Published:** 2011-10-19

**Authors:** You-Hong Jin, Motohide Takemura, Akira Furuyama, Norifumi Yonehara

**Affiliations:** ^1^Department of Anatomy, Affiliated Stomatological Hospital of Nanchang University, Jiangxi Province, Nanchang 330006, China; ^2^Department of Oral Anatomy and Neurobiology, Graduate School of Dentistry, Osaka University, Suita, Osaka 565-0871, Japan; ^3^Department of Oral Physiology, School of Dentistry, Ohu University, Koriyama, Fukushima 963-8611, Japan; ^4^Division of Dental Pharmacology, Department of Oral Medical Science, School of Dentistry, Ohu University, Koriyama, Fukushima 963-8611, Japan; ^5^Division of Pharmacology, School of Pharmaceutical Science, Ohu University, Koriyama, Fukushima 963-8611, Japan

## Abstract

Transient receptor potential vanilloid1 (TRPV1) and glutamate receptors (GluRs) are located in small diameter primary afferent neurons (nociceptors), and it was speculated that glutamate released in the peripheral tissue in response to activation of TRPV1 might activate nociceptors retrogradely. But, it was not clear which types of GluRs are functioning in the nociceptive sensory transmission. In the present study, we examined the c-Fos expression in spinal cord dorsal horn following injection of drugs associated with glutamate receptors with/without capsaicin into the hindpaw. The subcutaneous injection of capsaicin or glutamate remarkably evoked c-Fos expression in ipsilateral sides of spinal cord dorsal horn. This capsaicin evoked increase of c-Fos expression was significantly prevented by concomitant administration of MK801, CNQX, and CPCCOEt. On the other hand, there were not any significant changes in coinjection of capsaicin and MCCG or MSOP. These results reveal that the activation of iGluRs and group I mGluR in peripheral afferent nerves play an important role in mechanisms whereby capsaicin evokes/maintains nociceptive responses.

## 1. Introduction

Glutamate which is a major excitatory neurotransmitter in the central nervous system has been shown recently to play an important role in peripheral nociceptive transmission [1]. Concerning the existence of glutamate in the small-diameter afferent fibers, it has been reported that electrical stimulation of the sciatic nerve or noxious heat stimulation (50°C) caused an increase of the glutamate level in subcutaneous perfusate [2, 3].

Glutamate receptors (GluRs) are classified into ionotropic GluRs (iGluRs: NMDA, AMPA/kainate receptor) and metabotropic GluRs (mGluRs: group I, II, and III mGlu receptor). There is evidence that both iGluRs [4] and mGluRs [5] have been located in the peripheral processes of primary unmyelinated afferents. In this connection, electron microscope studies demonstrate that GluRs are transported from the DRG cell bodies into central and/or peripheral primary afferent terminals [6]. NMDA, AMPA, and kainate receptors (NMDA/AMPA-kainate receptors) are localized on unmyelinated axons at the dermal-epidermal junction in the glabrous and hairy skin of rats [7, 8] and human hairy skin [9]. The immunostain labeled unmyelinated fibers are assumed to be sensory fibers and not sympathetic efferents that are also unmyelinated. 

Besides these anatomical data, behavioral evidences support a role for peripheral GluRs in normal nociceptive transmission. Intraplantar injection of L-glutamate or iGluR agonists into the hindpaw evokes thermal and mechanical hyperalgesia and allodynia, which can be blocked by appropriate antagonists [7, 10–12]. 

Transient receptor potential Vanilloid 1 (TRPV1) is also located in small- and medium-sized dorsal root ganglion (DRG) neurons. A broad range of stimuli such as noxious heat, protons, lipid-derived endovanilloids, and inflammatory mediators either directly activate or modulate TRPV1 [13, 14], and stimulation of TRPV1 elicits the release of glutamate [12, 15, 16]. At the peripheral terminals of primary afferents, TRPV1-mediated Ca^2+^ influx triggers the release of neuropeptides and neurotransmitters, which is responsible for nociceptive processing [14]. TRPV1-expressing dorsal horn neurons in the spinal cord are revealed to be glutamatergic [17].

With regard to the interaction between TRPV1 and GluRs, there is some evidence supporting the modulation of TRPV1 function through GluRs. For example, group I mGluRs (especially mGluR5) are expressed together with TRPV1 in DRG neuron and increase thermal sensitivity by enhancing TRPV1 function at peripheral endings and central presynaptic terminals of nociceptors [18, 19]. Group II mGluRs are also coexpressed with TRPV1 and activation of mGluRs can inhibit nociceptive transmission induced by TRPV1 activation [20]. Furthermore, it was reported that the activation of peripheral iGluRs (NMDA receptors) may be important in the mechanisms whereby capsaicin evokes nociceptive trigeminal responses [21].

We also observed that GluRs, in particular, iGluRs and group I mGluR existing in peripheral endings of capsaicin-sensitive afferent fibers, play an important role in the development and maintenance of hyperalgesia following excitation of TRPV1 by determining the changes of glutamate levels in the extracellular space of rat hindpaw and pain behavior in the thermal withdrawal latency [12]. 

In this study, we are examining c-Fos levels concerning the neural activity in the spinal cord in response to peripheral activation of TRPV1, GluRs, or both in an attempt to see which GluRs drive highest levels of activity.

## 2. Materials and Methods

### 2.1. Experimental Procedures

All surgical and experimental procedures for animals were reviewed and approved by the Osaka University Faculty of Dentistry and the Ohu University Intramural Animal Care and Use Committees, and conformed to the guidelines of the International Association for the Study of Pain [14].

Adult male Sprague Dawley rats weighing 200 g–300 g (CLEA Japan, Inc. Tokyo) were used in all experiments. Rats were kept in a 12 h light/dark cycle and received food and water ad libitum.

### 2.2. Drug Administration

While animals were inside the small cage, drugs were administrated subcutaneously (s.c.) into the left hindpaw. 

Drugs were administered in the volume of 50 *μ*L, s.c., into the plantar surface of the left hindpaw using a 100 *μ*L Hamilton syringe (Reno, Nev, USA) with a 30-gauge needle without anesthesia when animals were kept in a small cage to hold them. The needle was inserted into the plantar skin proximal to the midpoint of the hindpaw. The applied concentrations of L-glutamate and GluR antagonists were decided based on the result obtained from the previous study [12].

### 2.3. c-Fos Immunohistochemistry

Fos immunoreactivity (labeled nuclei of Fos-immunoreactive spinal neurons) ipsi- and contralateral to the stimulated side was detected according to a standard avidin–biotin–peroxidase technique [18, 22]. Briefly, two hours after the drug injection, animals were deeply anesthetized with sodium-pentobarbital and perfused transcardially with 100 mL of 0.9% saline followed by 500 mL of 4% paraformaldehyde in 0.1 M phosphate buffer (PB; pH 7.4) and the spinal cord was taken out, postfixed in the same fixative overnight at 4°C, and then immersed into 20% sucrose in 0.1 M PB at 4°C until it sank. Serial transverse 60–80 *μ*m thick sections at L4-L6 were cut using a freezing microtome and collected in 0.02 M phosphate buffered saline (PBS). Sections were washed in PBS for 30 min and blocked with 1% normal goat serum for 30 min and then incubated in a rabbit antibody against c-Fos (1 : 7000 dilution; Santa Cruz Biotech, Santa Cruz, Calif, USA) for 60 min in room temperature and then for 12 h at 4°C. After washing in PBS for 30 min, sections were incubated in biotinylated goat antirabbit antiserum, and washed in PBS for 30 min and then immunohistochemically stained for 60 min using avidin-biotin-peroxidase complex (Vectastain, Vector Laboratories, Burlingame, Calif, USA). To visualize peroxidase activity, sections were immersed in 0.05% diaminobenzidine tetrahydrochloride, 0.1% ammonium nickel sulfate, and 0.01% hydrogen peroxide in 0.05 M Tris-HCl buffer (pH 7.2). Sections were washed in PBS for 30 min and then mounted on gelatin-coated slides, air-dried, and coverslipped.

### 2.4. Quantification of Number of c-Fos-Immunoreactive (ir) Cells

c-Fos-ir cells of laminae I-II and laminae III-IV of all sections (about 80–100) were counted in accordance with previously described methods [23]. In brief, among them, the best labeled 10 sections of the spinal cord segments from L4–L6 were chosen for the mean number per certain segment of labeled neurons. 

 Our quantification dealt only with the number and location of cell profiles with histologically detectable Fos immunoreactivity, where the intensity of positive cell profiles was not considered. The number of c-Fos-ir cell profiles in the spinal cord dorsal horn was counted only in laminae I-II and laminae III-IV, respectively, according to the method by which the dorsal horn was separated into two areas as follows: the lateral one-fourth of laminae I-II that is referred to as the posterior cutaneous (PC) territory in conjunction with the medial three-fourth of laminae I-II that corresponds to the terminal field of C fibers of primary neurons innervating the sciatic nerve, and laminae III-IV that is the terminal field of *A*_*β*_ fibers of primary neurons, [24, 25]. In all these tests, a double blind procedure was used to prevent the observers from knowing the experimental groups.

### 2.5. Drug Preparation

We used L-glutamate as GluR agonist. As GluR antagonists, the following drugs were used: selective noncompetitive NMDA-receptor antagonist, (5S,10R)-(+)-5-methyl-10,11-dihydro-5H-dibenzo[a,d]cyclo-hepten-5,10-imine hydrogen maleate [(+)-MK-801 hydrogen maleate]; competitive kainite/AMPA-receptor antagonist, 6-cyano-7-nitroquinoxaline-2,3-dione disodium (CNQX); group I mGluR selective noncompetitive mGlu1R antagonist, 7-(hydroxyimino) cyclopropa[b]chromen-1a-carboxylate ethyl ester (CPCCOEt); group II mGluR antagonist, (2S,3S,4S)-2-methyl-2-(carboxycyclopropyl)glycine (MCCG); selective group III mGluR antagonist, (R,S)-a-methylserine-O-phoephate (MSOP). These agonist and antagonists of the GluR were obtained from Tocris (Ballwin, MO, USA). 8-Methyl-N-vanillyl-6-noneamide (capsaicin) and N-[2-(4-chlorophenyl)ethyl]-1,3,4,5-tetrahydro-7,8-dihydroxy-2H-2benzazepine-2-carbothio-amide (capsazepine) were obtained from Sigma Chemical Co. (St. Louis, MO, USA), and Cayman Chemical (Ann Arbor, MI, USA), respectively. All the other chemicals were obtained from Wako Pure Chemical Industries, Ltd. (Osaka).

In accordance with the product material safety data sheets, L-glutamate was diluted in NaOH; MK801, MCCG, and MSOP were diluted in water. CNQX and CPCCOEt were diluted in dimethyl sulfoxide. The other drugs except for these were dissolved in saline. Capsaicin (Cap) was prepared as a 3 mM solution in saline containing 10% ethanol and 10% Tween 80. The pHs of all solutions were adjusted to 7.4.

### 2.6. Statistical Analysis

Statistical analysis of c-Fos-ir cells was performed by Student's *t*-test for unpaired values. All data was presented as a mean ± SEM. *P* values less than 0.05 or 0.01 were considered to indicate statistical significance.

## 3. Results

### 3.1. Time Course of Number of c-Fos-ir Cells in L4-L5 Spinal Dorsal Horn after Intraplantar Injection of Saline

**Figure 1:**
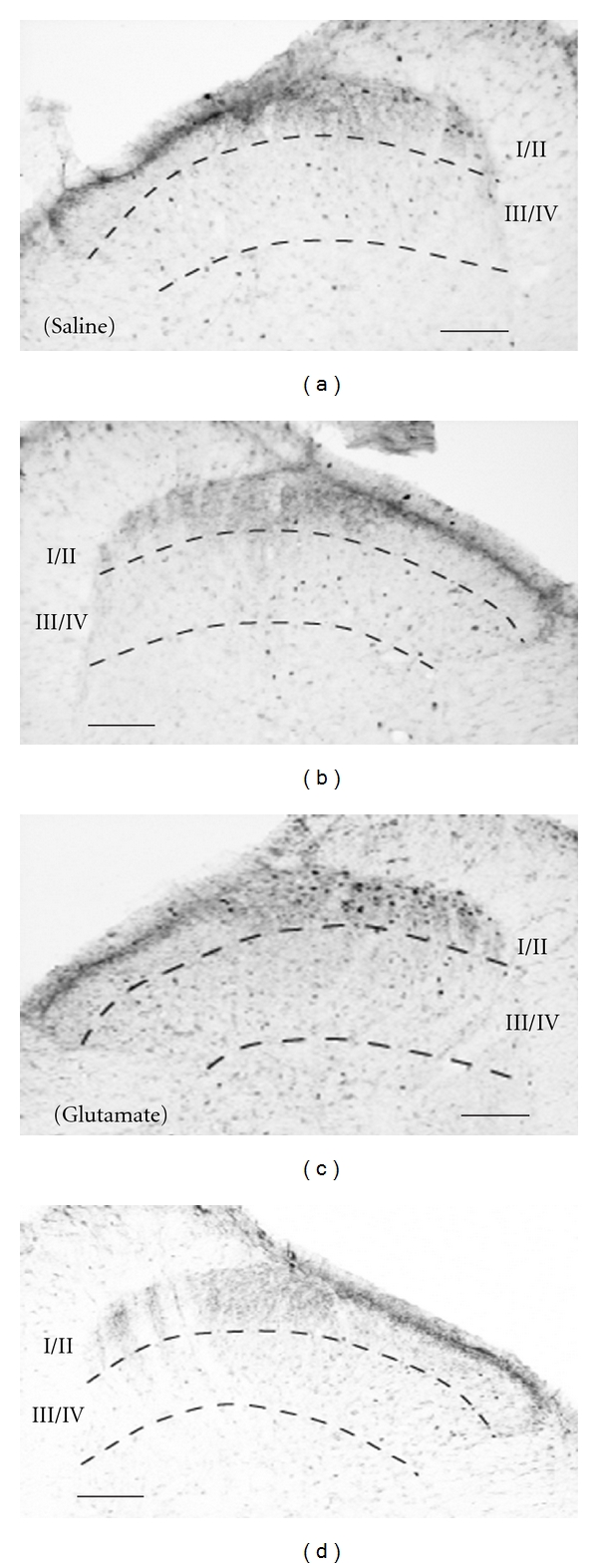
Photomicrographs showing saline-induced (a), (b) and glutamate-induced c-Fos-immunoreactive (ir) cells (c), (d) in the dorsal horn of L5 2 h after s.c. injection. (a) and (c) ipsilateral side. (b) and (d) contralateral side. Solid line indicates 100 *μ*m.

**Figure 2:**
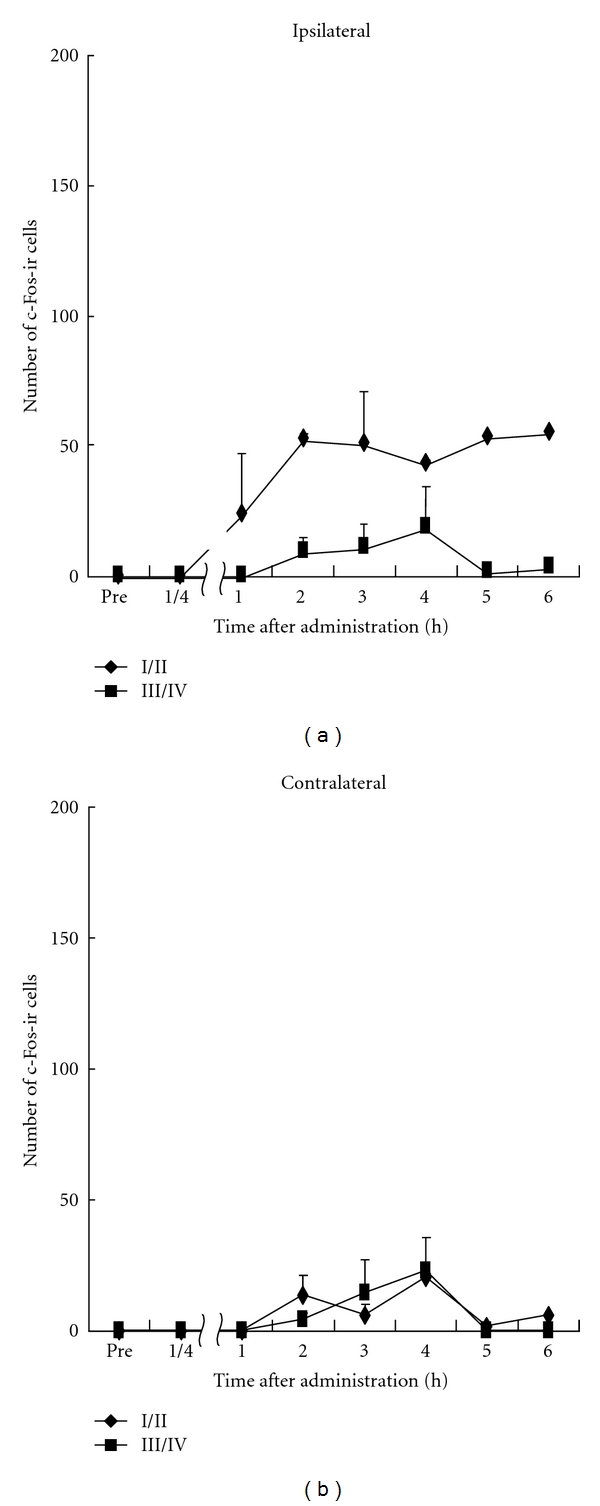
Time course of number of c-Fos-ir cells in the dorsal horn of L4-L5 after s.c. injection of saline. All data presented are mean ± SEM obtained from six animals.

Immunoreactivity for c-Fos appeared gray to black and homogeneously labeled the oval or roundish nucleus of cells in spinal dorsal horn at L5 (Figures 1, 5, and 6). In the intact animals, the basal level of c-Fos expression on the both sides of the lumbar spinal cord (L4-L5) was very low (zero or one c-Fos-ir cell per 60–80 *μ*m thick section), which was referred to as pretreatment value (pre). In animals administered with vehicle, c-Fos-ir cells were rarely distributed either in laminae I/II (60 ± 5) or in laminae III/IV (22 ± 8) on the ipsilateral side or on the contralateral side (I/II, 12 ± 4; III/IV, 7 ± 2) (Figure 2, Table 1).

### 3.2. Capsaicin- and Glutamate-Induced c-Fos Expression

In all the experimental tests with injection of capsaicin (3 mM) or glutamate (100 mM), the maximum number of labeled cells occurred consistently in laminae I and II (I/II) of the spinal dorsal horn on the ipsilateral side. 

**Figure 3:**
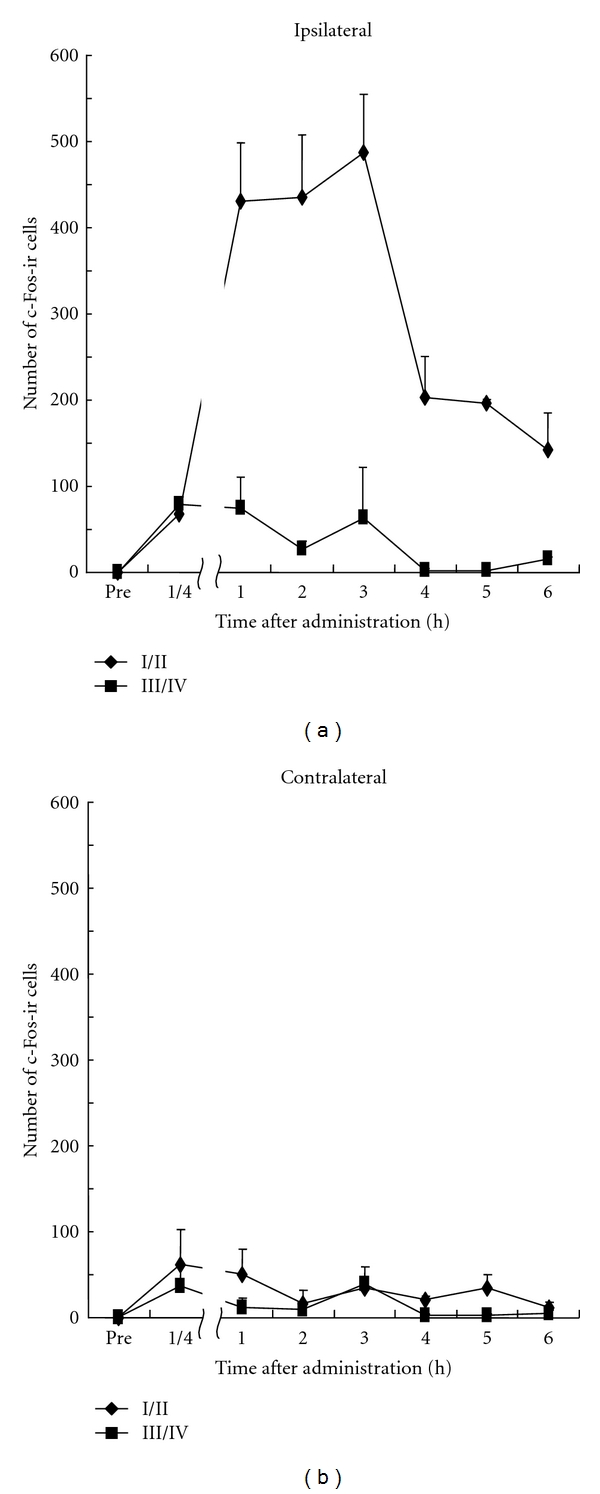
Time course of number of c-Fos-ir cells in the dorsal horn of L4-L5 after s.c. injection of capsaicin (3 mM). All data presented are mean ± SEM obtained from six animals.

The maximum number of c-Fos-ir cells (400–500) evoked by capsaicin was observed in laminae I/II on the ipsilateral side 1 h–3 h after injection, and much smaller number of c-Fos-ir cells (<100) occurred in laminae III and IV. This remarkable increase in laminae I/II on the ipsilateral side was maintained for more than 6 h. However, there was no difference between the numbers of c-Fos-ir cells in III/IV on the ipsilateral side and, that in laminae I/II and III/IV on the contralateral side at any measuring time (Figure 3, Table 1). 

**Figure 4:**
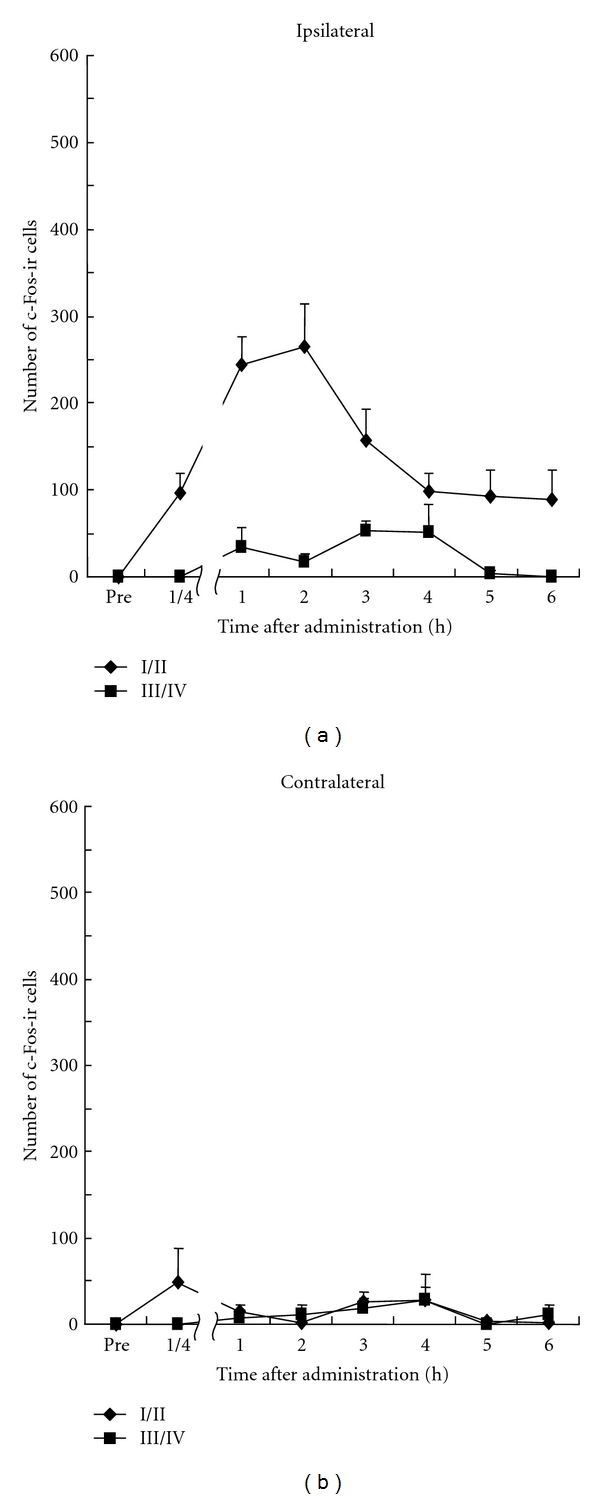
Time course of number of c-Fos-ir cells in the dorsal horn of L4-L5 after s.c. injection of glutamate. All data presented are mean ± SEM obtained from six animals.

In glutamate-treated animals, the increase of c-Fos-ir cells began 15 min after injection in laminae I/II on the ipsilateral side, and continued for 4 hours. The maximum number was observed (283 ± 18) 2 h after injection. The glutamate-induced c-Fos expression in laminae I/II and laminae III/IV on the ipsilateral side was lower than that with capsaicin. The numbers of c-Fos-immunopositive cells on the contralateral side was modest either with capsaicin (I/II, 44 ± 13; III/IV, 20 ± 9) or glutamate (I/II, 19 ± 8; III/IV, 12 ± 6) at each peak time on the ipsilateral side (Figure 4, Table 1). 

### 3.3. Effects of iGluRs Antagonists on the Capsaicin-Induced c-Fos Expression

To investigate which types of GluRs were involved in the peripheral mechanisms whereby capsaicin evokes/maintains nociceptive responses, the effects of GluRs antagonists on the capsaicin-induced c-Fos expression were examined at 2 h after treatment, because the maximum number of labeled cells was observed 1 h–3 h after capsaicin injection. 

**Figure 5:**
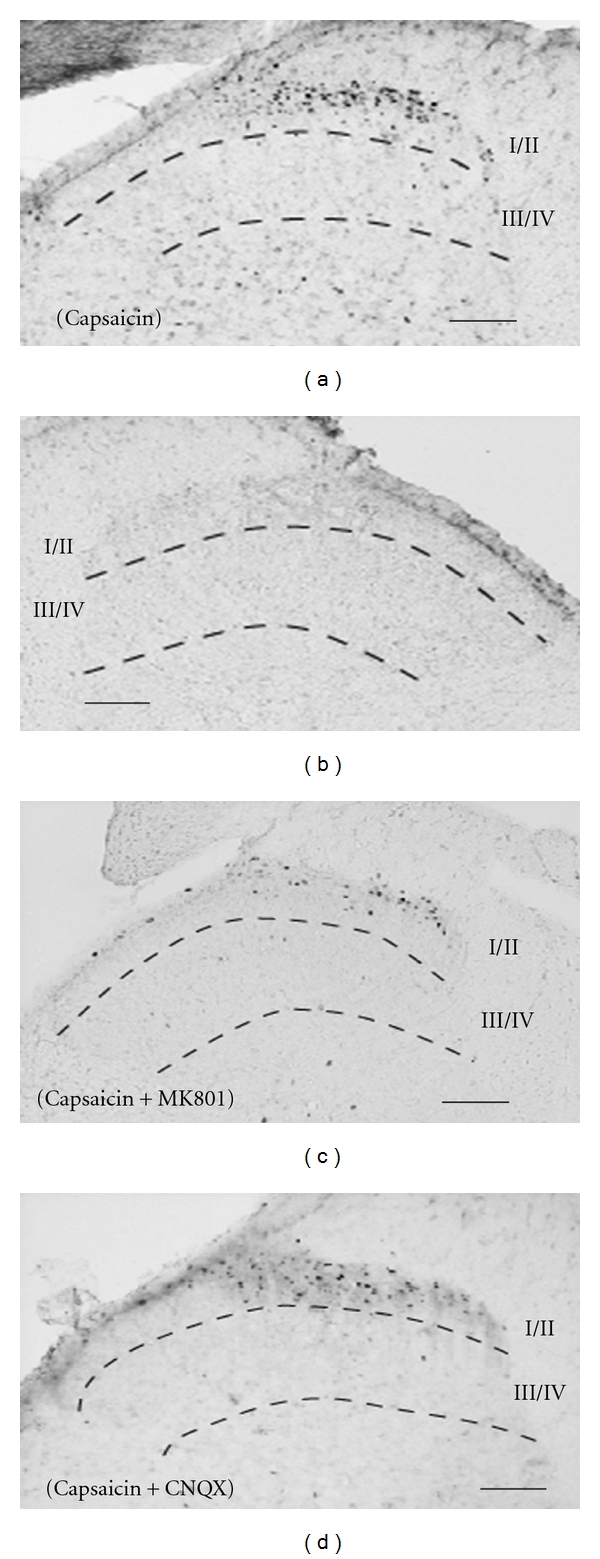
Photomicrographs showing capsaicin-induced c-Fos-ir cells in the dorsal horn of L5 2 h after s.c. injection of capsaicin alone (a), (b), or combined with MK801 ((c), 1 mM) or with CNQX ((d), 1 mM). All data presented are mean ± SEM obtained from six animals. (a), (c), and (d) ipsilateral side. (b) contralateral side. Solid line indicates 100 *μ*m.

Few c-Fos-ir cells were found in laminae I/II and laminae III/IV of the ipsilateral dorsal horn after each single injection of ionotropic glutamate receptors antagonists; MK-801 (1 mM) (I/II, 79 ± 3; III/IV, 11 ± 7) or CNQX (1 mM) (I/II, 70 ± 8; III/IV, 7 ± 3), similar to vehicle injection (I/II, 60 ± 5; III/IV, 22 ± 8). The numbers of capsaicin-induced c-Fos-ir cells in laminae I/II (489 ± 34), but not in laminae III/IV (63 ± 18), were significantly decreased, when MK801 and CNQX were injected with capsaicin (Cap + MK801, I/II, 227 ± 32, III/IV, 14 ± 4, Cap + CNQX, I/II, 205 ± 40, III/IV, and 11 ± 7) (Figure 5, Table 1). The numbers of capsaicin-induced c-Fos-ir cells on the contralateral sides did not significantly change by any of drugs with/without capsaicin.

### 3.4. Effects of mGluRs Antagonists on Capsaicin-Induced c-Fos Expression

**Figure 6:**
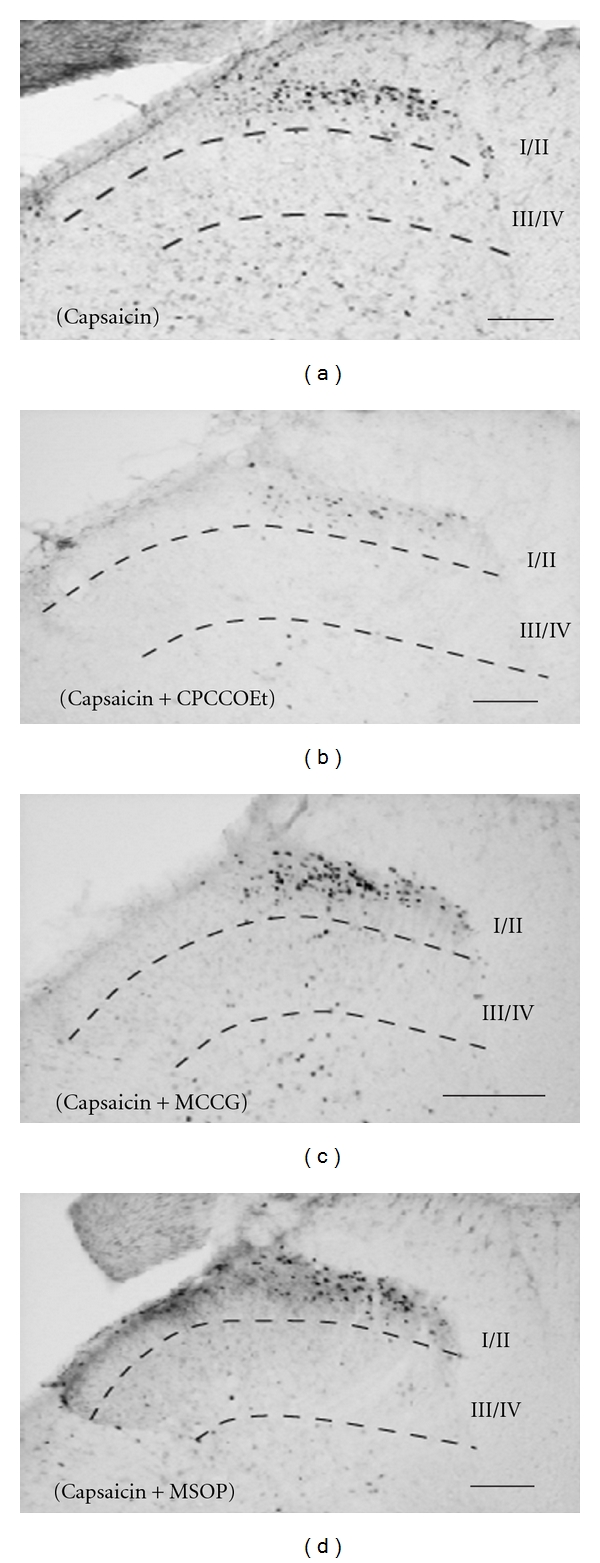
Photomicrographs showing capsaicin-induced c-Fos-ir cells in the dorsal horn of L5  h after s.c. injection of capsaicin alone (a), combined with CPCCOEt ((b), 5 mM), with MCCG ((c), 5 mM), and with MSOP ((d), 5 mM). All data presented are mean ± SEM obtained from six animals. (a), (b), (c), and (d) ipsilateral side. Solid line indicates 100 *μ*m.

Few c-Fos-ir cells in the ipsilateral laminae I/II and III/IV, and fewer cells in the contralateral sides, were observed with single injection of CPCCOEt (5 mM) (*I*/*II*, 59 ± 8, *II* *I*/*IV*, 1 ± 1), MCCG (5 mM) (I/II, 63 ± 10, III/IV, 3 ± 2), and MSOP (5 mM) (I/II, 66 ± 16, III/IV, 5 ± 3). Coinjection of CPCCOEt with capsaicin (Cap + CPCCOEt) significantly decreased the number of capsaicin-induced c-Fos-ir cells in the ipsilateral laminae I/II (236 ± 58), but not in laminae III/IV and contralateral laminae I/II and III/IV. There was no significant change in the number of c-Fos-ir cells in the ipsilateral laminae I/II, and III/IV by injection of MCCG combined with capsaicin (Cap + MCCG; I/II, 560 ± 85, III/IV, 27 ± 10) or by injection of MSOP combined with capsaicin (Cap + MSOP; I/II, 383 ± 21, III/IV, 22 ± 3) compared to single injection of capsaicin, respectively (Figure 6, Table 1).

## 4. Discussion

It has been previously reported that glutamate was released peripherally by electrical stimulation of the sciatic nerve, heat stimulation (50°C), or local application of capsaicin cream to the hind instep [2, 3]. High-dose repeated pretreatment with capsaicin leading to desensitization of small-diameter sensory neurons (nociceptors) [26–32], significantly attenuated the glutamate release.

Capsaicin significantly decreased thermal withdrawal latency to irradiation [12]. These effects of capsaicin were inhibited by not only pretreatment with capsazepine, but also coinjection of capsaicin with GluR antagonists (iGluRs and group I mGluR). These results suggest that glutamate is released from the peripheral endings of nociceptors by TRPV1 activation. Since DRG cells as well as their central and peripheral terminals express iGluRs (NMDA, AMPA/kainate receptor) and mGluRs (group I, II, and III mGluRs) [4–9, 17], released glutamate could activate peripheral iGluRs and group I mGluR in development and/or maintenance of nociception.

 In the present study, we examined the c-Fos expression as indicator of neuronal activity by pharmacological approach. To clarify the contribution of peripheral GluRs in development and/or maintenance of nociception, L4-L5 spinal dorsal horn neuronal activity, induced by intraplantar injection of drugs associated with glutamate receptors with/without capsaicin into the hindpaw, was investigated.

The subcutaneous injection of capsaicin or glutamate remarkably evoked c-Fos expression in ipsilateral sides of spinal cord dorsal horn and the maximum number of labeled cells occurred consistently in laminae I and II (I/II) of the spinal dorsal horn, where small-diameter DRG neurons terminate. The number of capsaicin-evoked c-Fos-immunoreactive (ir) cells was about twice as much as glutamate induced. A subset of capsaicin-sensitive nociceptors might express GluRs at the peripheral endings.

This capsaicin-evoked increase of c-Fos expression was significantly prevented by concomitant administration of MK801, CNQX, and CPCCOEt. These results were in agreement with the findings of the behavioral pharmacology, that is, capsaicin-induced thermal hypersensitivity was inhibited by the injection of the antagonists such as MK801, CNQX, or CPCCOEt in combination with capsaicin [12]. It should be noted that approximately 50% of the capsaicin-evoked c-Fos-ir cells were still positive, in spite of the complete inhibition of capsaicin-induced thermal hyperalgesia caused by injection of these antagonists [12]. On the other hand, there were not any significant changes in co-injection of capsaicin and MCCG or MSOP. 

In conclusion, some of the capsaicin-sensitive neurons projected in laminae I and II (I/II) of the spinal cord play an essential role in mechanisms whereby capsaicin evokes/maintains nociceptive responses, though the function of the other capsaicin-sensitive neurons remained unclear. Ionotropic glutamate receptors and/or group I mGluR at the afferent terminals of these nociceptive neurons might be required for capsaicin-induced hyperalgesia, because blocking them resulted in remarkable analgesia [12]. 

Our present study not only supports the view that GluRs existing in peripheral endings of capsaicin-sensitive afferent fibers are involved in the development and maintenance of hyperalgesia following excitation of TRPV1, but also suggests the probability of development of new medicines. Namely, the formulation of the peripheral iGluRs and group I mGluR antagonists that do not cross the blood brain barrier may be of potential benefit by reducing peripheral nociceptive excitability, and therefore they could provide a new therapeutic target to pain control in the periphery.

## Figures and Tables

**Table 1:** Mean value of c-Fos-ir cells in the dorsal horn of L4-L5 2 h after s.c. injection of capsaicin, glutamate, and glutamate receptors antagonists with/without capsaicin. The value in each group was represented mean ± SEM obtained from at least 10 animals, and the difference of the means was analyzed with the Student's *t*-test.

Group	Ipsilateral		Contralateral	
	I/II-layer	III∖IV-layer	I/II-layer	III∖IV-layer
Vehicle	60 ± 5	22 ± 8	12 ± 4	7 ± 2
Capsaicin(cap)	489 ± 34*	63 ± 18	44 ±13	20 ± 9
Glutamate	283 ± 18*	36 ± 5	19 ± 8	12 ± 6

MK801	79 ± 3	11 ± 7	33 ± 12	9 ± 5
CNQX	70 ± 8	7 ± 3	14 ± 7	3 ± 2
CPCCOEt	59 ± 8	6 ± 3	10 ± 4	6 ± 2
MCCG	63 ± 10	5 ± 2	28 ± 11	5 ± 2
MSOP	66 ± 16	5 ± 3	9 ± 4	5 ± 3

Cap + MK801	227 ± 32^#^	14 ± 4	8 ± 6	3 ± 2
Cap + CNQX	205 ± 40^#^	11 ± 7	22 ± 12	3 ± 2
Cap + CPCCOEt	236 ± 58^#^	17 ± 11	12 ± 7	4 ± 3
Cap + MCCG	560 ± 85	27 ± 10	24 ± 9	3 ± 1
Cap + MSOP	383 ± 21	22 ± 3	18 ± 13	4 ± 1

*Significant difference at *P* < 0.05 between vehicle and capsaicin, or glutamate-treated group. ^#^Significant difference at P < 0.05 between capsaicin and capsaicin + MK801, or capsaicin + CNQX, or capsaicin + CPCCOEt-treated group.
